# Aldehyde Dehydrogenase, a Marker of Normal and Malignant Stem Cells, Typifies Mesenchymal Progenitors in Perivascular Niches

**DOI:** 10.1093/stcltm/szad024

**Published:** 2023-06-01

**Authors:** Mario A Gomez-Salazar, Yiyun Wang, Neelima Thottappillil, Reef W Hardy, Manon Alexandre, Fabian Höller, Niall Martin, Zaniah N Gonzalez-Galofre, Dorota Stefancova, Daniele Medici, Aaron W James, Bruno Péault

**Affiliations:** Center for Regenerative Medicine and Center for Cardiovascular Research, University of Edinburgh, Edinburgh, UK; Department of Pathology, Johns Hopkins University, Baltimore, MB, USA; Department of Pathology, Johns Hopkins University, Baltimore, MB, USA; Department of Pathology, Johns Hopkins University, Baltimore, MB, USA; Orthopaedic Hospital Research Center and Broad Stem Cell Research Center, David Geffen School of Medicine, University of California, Los Angeles, CA, USA; Center for Regenerative Medicine and Center for Cardiovascular Research, University of Edinburgh, Edinburgh, UK; Polytech Marseille, Aix Marseille University, Marseille, France; Center for Regenerative Medicine and Center for Cardiovascular Research, University of Edinburgh, Edinburgh, UK; Center for Regenerative Medicine and Center for Cardiovascular Research, University of Edinburgh, Edinburgh, UK; Center for Regenerative Medicine and Center for Cardiovascular Research, University of Edinburgh, Edinburgh, UK; Center for Regenerative Medicine and Center for Cardiovascular Research, University of Edinburgh, Edinburgh, UK; Center for Regenerative Medicine and Center for Cardiovascular Research, University of Edinburgh, Edinburgh, UK; Department of Pathology, Johns Hopkins University, Baltimore, MB, USA; Center for Regenerative Medicine and Center for Cardiovascular Research, University of Edinburgh, Edinburgh, UK; Orthopaedic Hospital Research Center and Broad Stem Cell Research Center, David Geffen School of Medicine, University of California, Los Angeles, CA, USA

**Keywords:** *tunica adventitia*, aldehyde dehydrogenase, mesenchymal stem cell, mesenchymal stromal cell, pericyte, stem cell niche

## Abstract

Innate mesenchymal stem cells exhibiting multilineage differentiation and tissue (re)generative—or pathogenic—properties reside in perivascular niches. Subsets of these progenitors are committed to either osteo-, adipo-, or fibrogenesis, suggesting the existence of a developmental organization in blood vessel walls. We evaluated herein the activity of aldehyde dehydrogenase, a family of enzymes catalyzing the oxidation of aldehydes into carboxylic acids and a reported biomarker of normal and malignant stem cells, within human adipose tissue perivascular areas. A progression of ALDH^Low^ to ALDH^High^ CD34^+^ cells was identified in the *tunica adventitia*. Mesenchymal stem cell potential was confined to ALDH^High^ cells, as assessed by proliferation and multilineage differentiation in vitro of cells sorted by flow cytometry with a fluorescent ALDH substrate. RNA sequencing confirmed and validated that ALDH^High^ cells have a progenitor cell phenotype and provided evidence that the main isoform in this fraction is ALDH1A1, which was confirmed by immunohistochemistry. This demonstrates that ALDH activity, which marks hematopoietic progenitors and stem cells in diverse malignant tumors, also typifies native, blood vessel resident mesenchymal stem cells.

## Graphical Abstract

**Figure:**
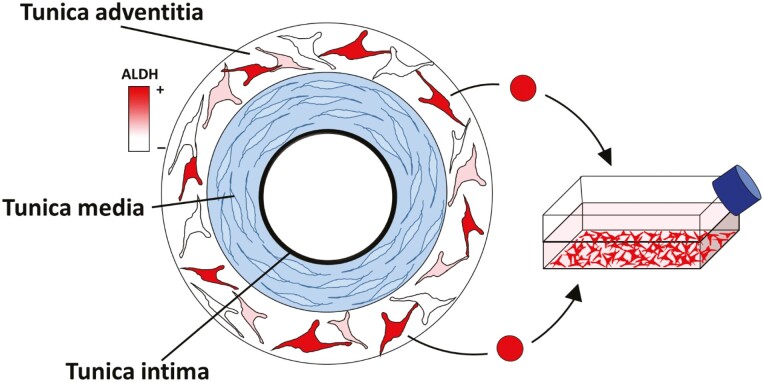
The *tunica adventitia*, which ensheathes arteries and veins, contains progenitor cells displaying high-ALDH activity that upon purification and culture turn into mesenchymal stem cells.

## Introduction

Blood vessels harbor presumptive mesenchymal stem/stromal cells (MSCs), as reflected by the growth of MSCs in cultures of all dissociated vascularized tissues.^1,2^ Two known perivascular cell types give rise to human MSCs in vitro: pericytes, in direct contact with endothelial cells in capillaries and micro vessels, and cells localized in the *tunica adventitia*, at the outmost periphery of larger blood vessels.^3,4^ Cell lineage tracing in mice has revealed the participation of perivascular cells in the regeneration of adipose tissue,^5^ skeletal muscle,^6^ dental pulp,^7^ bone,^8^ and follicular dendritic cells,^9^ as well as in pathologic fibrosis^10,11^ and atherosclerosis.^12^ Such tissue resident mesodermal progenitors are diverse: marker defined discrete subsets of perivascular cells possess superior, or exclusive, potentials for osteogenesis,^13-15^ adipogenesis,^16^ and fibrosis.^12,17-20^

Aldehyde dehydrogenase (ALDH), a family of 19 enzyme isoforms, catalyzes the oxidation of aldehydes into carboxylic acids. High-ALDH activity has been documented in normal hematopoietic stem cells (HSCs) and in malignant progenitors for acute myeloid leukaemia, melanoma, and ovarian, brain, prostate, and breast cancer.^21-32^ ALDH being intracellular, colorimetric assays of activity, such as ALDEFLUOR, have been used to identify and isolate normal and tumoral human stem cells by flow cytometry.^26,33,34^ Many isoforms of ALDH can metabolize the ALDEFLUOR substrate, which complicates the identification of cell-specific enzyme subtypes. Zhou and colleagues analyzed the contribution of the 19 ALDH isoforms to the ALDH^High^ cell compartment in cancer cell lines and found 9 of these to be detected by the ALDEFLUOR assay.^35^ The ALDH1 subfamily is prominently associated with stem cell properties and drug resistance in cancer.^22,27,29,36-39^ ALDH1 enzymes are required for the biosynthesis of retinoic acid (RA) in the cytosol. In specific, the ALDH1A1 isoenzyme demonstrates high affinity to oxidize retinaldehyde (retinal) into RA, in comparison to other ALDH isoforms, and is, therefore, more involved in gene regulation,^40^ although ALDH1A2 and ALDH1A3 can also metabolize retinal.^36^

We previously described the existence of ALDH^High^ and ALDH^Low^ subsets of native human perivascular cells that were further defined by transcriptome analysis. As a proof of concept, gene coregulation analysis showed that FACS-purified ALDH^High^ perivascular cells are developmentally more primitive than ALDH^Low^ counterpart cells.^41^ Here, we characterized adventitial cells with high-ALDH activity, showing that this subset evokes a distinctive mesenchymal stem cell type once explanted in culture. In fine, combined immunohistochemistry and bulk- and single-cell RNA sequencing identified high-ALDH1A1 activity as a novel marker of tissue resident, presumptive mesenchymal stem cells.

## Methods

### Isolation of Perivascular Cells From Human Adipose Tissue

Adipose tissue was collected from female volunteers undergoing cosmetic liposuction. Informed consent from the donor was signed, and approval was granted by the South East Scotland Research Ethics committee (Ref number: 16/SS/0103). The tissue was next processed according to our established protocol.^3,4^

Briefly, the lipoaspirate was digested with 1 mg/mL type II collagenase (Gibco, Grand Island, NY) in Dulbecco’s Modified Eagle’s Medium (DMEM) containing 0.5% bovine serum albumin (Sigma-Aldrich, St. Louis, MO) at 37 °C for 60 minutes under agitation, followed by centrifugation to remove adipocytes. The digested tissue was filtered using strainers (100 μm and 70 μm pore size) and washed with 2% fetal calf serum (FCS) in PBS (v/v). The cell pellet was resuspended and incubated in red cell lysis buffer (155 mM NH_4_Cl, 10 mM KHCO_3_, and 0.1 mM EDTA) at RT for 10 minutes. After centrifugation, the stromal vascular fraction (SVF) was resuspended in PBS and filtered at 40 μm. Next, the SVF was washed and stained with the ALDEFLUOR kit for 45 minutes at 37 °C according to manufacturer’s instructions (Stem Cell Technologies, Vancouver, Canada). Cells were then resuspended in ALDEFLUOR buffer and stained with the following antibodies: CD31-V450 (1:400), CD34-PE (1:100), CD45-V450 (1:400), and CD146-BV711 (1:100) (all from BD Biosciences, San Jose, CA). Pericytes (CD146+, CD34−, CD31−, CD45−) and adventitial cells (CD34+, CD146−, CD31−, CD45−) were sorted using a BD FACS FUSION sorter (BD Biosciences, San Jose, CA) based on the marker combinations previously described.^2,3^ Further analysis of flow cytometry data was performed using Flowjo software.

### Fluorescent Immunohistochemistry

Human fat tissue was embedded in optimal cutting temperature compound (OCT) (Sakura, Torrance, CA). Embedded samples were stored at −80°C and cryosectioned at 8-10 μm thickness. Sections were fixed in a solution of 1:1 acetone/methanol prior to staining. Nonspecific antibody binding was blocked with 10% goat serum in PBS (Sigma-Aldrich) for 1 hour at RT. The following lectin and primary antibodies were used: directly biotinylated *Ulex europaeus* lectin (UEA-1) (1:200; Vector-B1065, Vector Laboratories, Burlingame, CA), CD34 (1:100; ab139551, Abcam, San Francisco, CA), ALDH1A1 (1:100; 22109-1-AP, Proteintech, Manchester, UK) and incubated at 4 °C overnight. After washing with PBS, sections were incubated for 1 hour at RT with species-specific secondary antibodies diluted 1:300. The following fluorochrome-conjugated secondary antibodies were used: anti-mouse-Alexa 555 IgG, anti-rabbit-Alexa 647 IgG, and streptavidin conjugated 488 (all from Life Technologies, Gaithersburg, MD). Slides were mounted using Fluoramount G containing DAPI (SouthernBiotech, Birmingham, AL), and images were acquired using an epifluorescence microscope (Zeiss Observer, Zeiss, Oberkochen, Germany; Olympus BX61, Olympus, Tokyo, Japan). Images were processed using Fiji software or ZEN Blue lite version (Zeiss).

For paraffin-embedded sections, slides were dewaxed by sequential incubations with xylene. After dewaxing, slides were boiled in citrate buffer (10mM sodium citrate,0.05% Tween, pH 6) for 5 minutes for antigen retrieval.

### Cell Culture

Sorted perivascular cells were seeded and cultured (37°C, 5% CO_2_) in Endothelial Cell Growth Medium (EGM-2 Bulletkit-Lonza, Gaithersburg, MD) in plates precoated with 0.1% gelatin. Once confluent, cells were passaged into basal medium consisting of DMEM Glutamax (Gibco) supplemented with 100 μg/mL streptomycin (Sigma-Aldrich), 100 U/mL penicillin (Sigma-Aldrich), and 20% FCS (Sigma-Aldrich). Cells were used for experiments between passages 4 and 5.

For assessment of ALDH activity in vitro, the ALDEFLUOR assay was used following manufacturer’s instructions.

### Proliferation

To determine proliferation based on cell count, cells were seeded at a density of 14 000 cells per well in triplicate in a 24-well plate, and then counted using a Thermo Fisher Countess II Automated Cell Counter (Thermo Fisher, Waltham, MA). For selected studies, alamarBlue (Thermo Fisher) or MTS assay (Promega Corporation, Madison, WI) were used to assess proliferation, following the manufacturer’s instructions.

### Differentiation Assays

For adipogenic differentiation, cells were cultured in medium composed of DMEM, 10% FCS, 1 μM dexamethasone, 0.5 mM isobutylmethylxanthine, 200 μM indomethacine, and 10 μM insulin (all from Sigma-Aldrich) for 14 days. Cells were fixed with 4% PFA (paraformaldehyde), washed with 70% ethanol, and incubated with Oil Red O (Sigma-Aldrich) for detection of lipids.^3,4^ For quantification of adipogenesis, the area covered by adipocytes was measured. For certain experiments, photometric quantification was performed by adding isopropanol to the stained cells to extract Oil Red O, which was then analyzed at 540 nm.

For osteogenic differentiation, cells were cultured in medium composed of DMEM, 10% FCS, 0.1 μM dexamethasone, 50 μM ascorbate-2-phosphate, and 10 mM β-glycerophosphate (Sigma-Aldrich). Cells were fixed with 4% PFA, washed with milliQ water and incubated in alizarin red (Sigma-Aldrich) solution for 15 minutes at RT for detection of calcium.^3,4^ For quantification, alizarin red staining was measured by photometric quantification. Stained cells were treated with sodium hydroxide (0.1 N) to extract alizarin red, which was then analyzed at 548 nm.

### Bulk and Single-Cell RNA Sequencing

RNA of ALDH-high and ALDH-low subsets of adventitial cells was extracted using the Qiagen RNeasy Micro kit (Qiagen, Germantown, MD). Total RNA was quantified and measured for RNA integrity numbers of 7.7 or greater (2100 Agilent Bioanalyzer, Santa Clara, CA). For each subpopulation, complementary DNA (cDNA) was then prepared by using the NuGen’s Ovation RNA-Seq System V2 kit from ∼100 ng of RNA pooled from 2 or 3 donors and used as input for Ovation’s Ultralow DR Multiplex System 1-8 (Nugen, San Carlos, CA). High-throughput sequencing was conducted on an Illumina Hiseq 2000 platform to generate 100-bp paired-end reads at a depth of coverage of ∼79 million reads. Cluster formation achieved ∼86%, whereas the quality of reads exceeded 95%. Analysis was performed using PARTEK FLOW software (Partek Inc., Chesterfield, MO).

scRNA-seq data were obtained from the Gene Expression Omnibus (GEO) repository, accession number GSE128889 (GSM3717979, GSM3717977).^42^ Initial quality control selected cells expressing >200 and <4000 genes and a mitochondrial content <8%. Data normalization, dimensional reduction, and clustering were conducted in Seurat (RRID:SCR_016341) as previously described in the original publication except for altered clustering resolutions. Next, clusters expressing markers of hematopoietic cells (*CD45*), endothelial cells (*PECAM1, CHD5*), and pericytes (*ACTA2, PDGFRβ*) were removed, whereas clusters expressing CD34 and PDGFRα were identified as adventitial cells and kept for downstream analysis. Subsequently, we subset subpopulations from these adventitial cells expressing values of ALDH1A1 >1.5 (HIGH) and ALDH1A1 <0.5 (LOW/NEG) and merged the new subsets into one. Analysis of functional enrichment was performed using Metascape.^40^

### Statistical Analysis

Statistical analyses were performed by GraphPad Prism (RRID: SCR_002798, Version 7.0). The Shapiro-Wilk normality test was used to analyze all data sets. Unpaired 2-tailed Student *t* tests were used for 2-sample comparisons (2-sided). When specified, Welch’s correction was used. Two-way ANOVA test was used for comparisons of 2 with different time points, followed by Bonferroni’s multiple comparisons test. *P <.05 and **P < .01 were considered significant.

## Results

### Distribution of ALDH Activity in Human Adipose Tissue Shows Enrichment in Vascular Adventitial Cells

**Figure 1.**
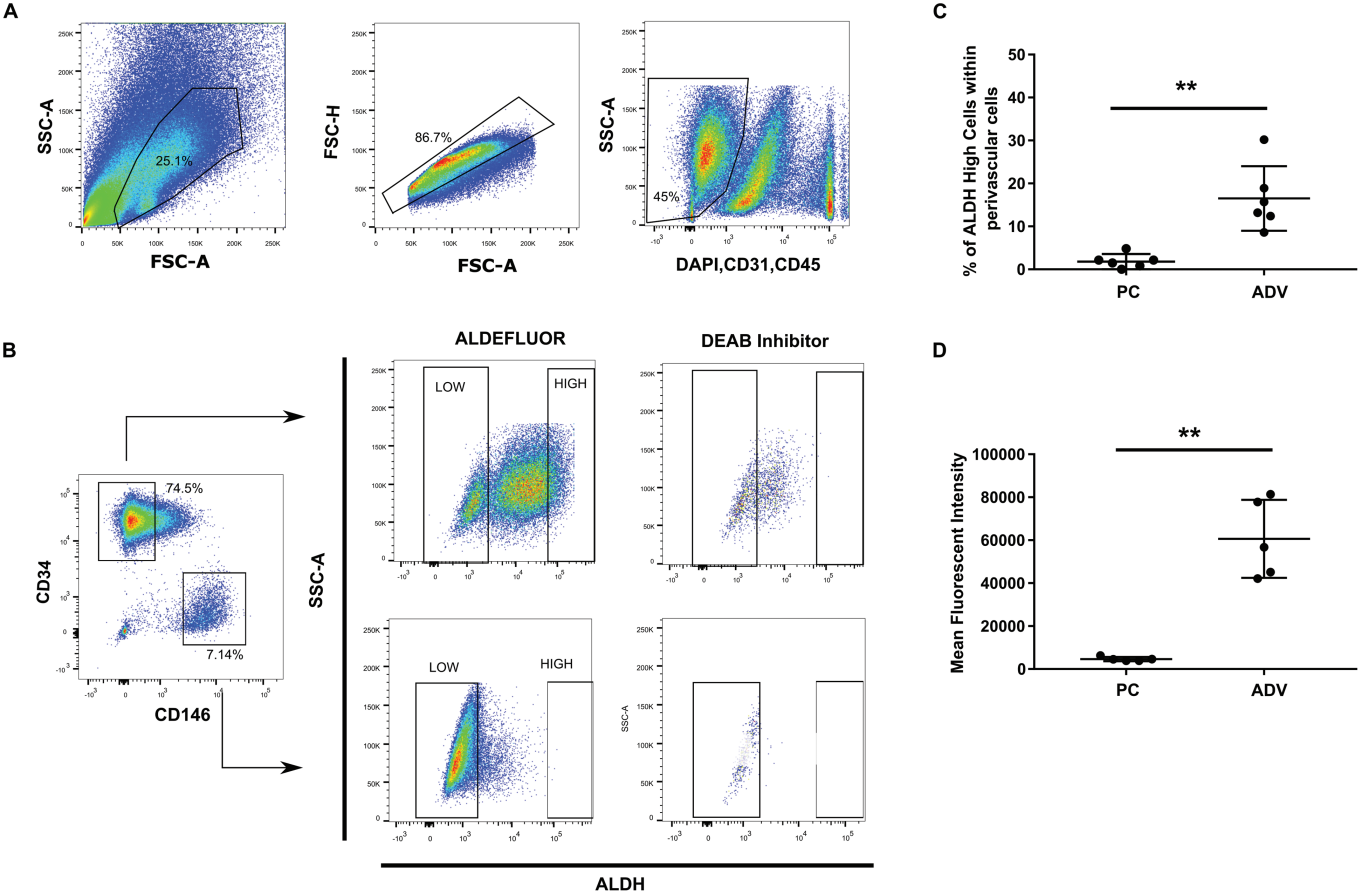
Human perivascular cell isolation from white adipose tissue by flow cytometry according to ALDH activity. (**A**) Perivascular cells are identified as live single cells, negative for CD31, CD45, CD56. (**B**) Adventitial cells and pericytes were gated on expression of CD34 and CD146, respectively. High-ALDH activity was identified using the DEAB inhibitor. (**C**) Quantification of ALDH^High^ pericytes (PC) and adventitial cells (ADV). (**D**) Quantification of ALDH activity using the ALDELFLUOR assay on pericytes and adventitial cells. Unpaired *t*-test with Welch’s correction was used. Data are shown as mean ± SD. *n* = 6. **P < .01.

Perivascular cells, ie, pericytes and adventicytes, with low-/high-ALDH activity were identified and isolated from adipose tissue by fluorescence-activated cell sorting (FACS) using the ALDEFLUOR fluorescent ALDH substrate, as previously described.^2,37^ Briefly, the fat SVF was stained with antibodies against CD31, CD45, CD146, and CD34. After negative selection of endothelial and hematopoietic cells on CD31 and CD45 expression, pericytes and adventitial cells were identified by the expression of CD146 and CD34, respectively. Subsequently, cells with high-ALDH activity were identified using the competitive inhibitor of ALDH enzymes N,N-diethylaminobenzaldehyde (DEAB) (Fig. 1B); DEAB is a potent inhibitor of cytosolic ALDH (ALDH1), used as a control to distinguish cells with enzymatic activity. The distribution of ALDH activity was observed to be totally different between these 2 cell subsets. The fraction of cells exhibiting detectable ALDH activity is much smaller among pericytes than in adventitial cells, and virtually no ALDH^High^ pericytes are present, whereas ALDH high activity comprised 15% of adventicytes (Fig.1C). Globally, adventitial cells have much higher ALDH activity (60 639 ± 8130) compared to pericytes (4648 ± 448.6), as assessed by fluorescence quantification (Fig. 1D), supporting the notion that adventitial cells have stem cell characteristics.^21^

### High-ALDH Activity Marks a Population of Adventitial Cells With MSC Potential

**Figure 2.**
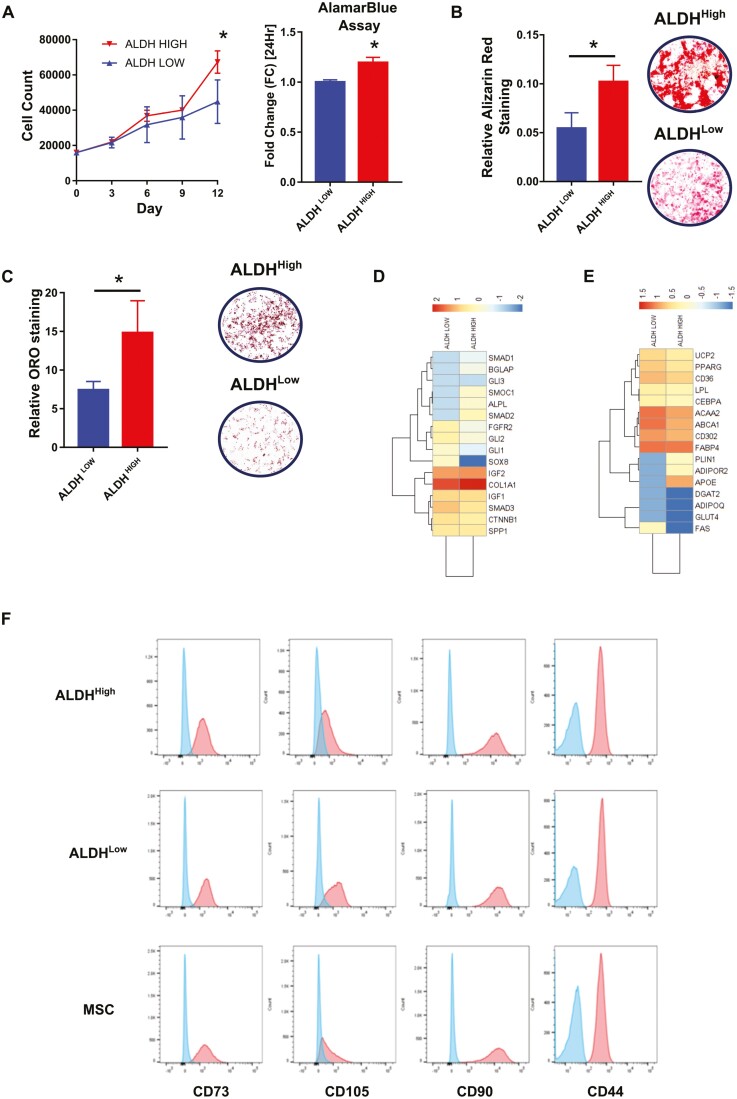
Functional characterization in culture of ALDH subsets of adventitial cells. ALDH^High^ and ALDH^Low^ subsets of adventitial cells were purified from adipose tissue as described in Fig. 1 and immediately placed in culture. (**A**) Cells were counted at different time points to assess proliferation rates, in paralallel an AlamarBlue assay was performed to analyze proliferation at 48 hour. (**B**) After seeding in relevant conditions, osteogenic differentiation was assessed by alizarin red staining, then measured by photometric quantification of alizarin red. (**C**) Similarly, adipogenesis was assessed by Oil Red O (ORO) staining and quantified by measuring the area covered by adipocytes. (**D**) Expression of osteogenesis related genes. (**E**) Expression of adipogenesis related genes. (**F**) Flow cytometry detection of canonical MSC markers in cultured ALDH subsets of adventitial cells. Unpaired *t*-test with Welch’s correction was used. Two-way Anova was used for proliferation at different time points .Data are shown as mean ± SD. *n* = 6. *P ≤ .05.

On the premise that adventitial cells show stem cell properties, based on ALDH activity, we focused our attention on this perivascular cell type. For in vitro characterization, we used subsets of adventicytes sorted by FACS from the extremes of the ALDH activity spectrum (low and high activity) to prevent contamination between fractions (Fig. 1B), which were cultured for 4 to 5 passages. Cell counts at successive time points and Alamar blue (a cell viability reagent) staining at 24 hour demonstrated that ALDH^High^ adventitial cells divide at a higher rate in culture compared to the ALDH^Low^ fraction (Fig. 2A). It is worth mentioning that cells with high-ALDH activity start proliferating 2 days after isolation, whereas cells from the ALDH low fraction took up to 10 days to attach and start dividing. Moreover, only ALDH^High^ adventitial cells could form colonies (CFU-F assay) after single cell index FACS sorting, with 30% efficiency of colony formation, while ALDH^Low^ cells were not able to form colonies (0%). Furthermore, we evaluated ALDH activity following in vitro expansion by seeding ALDH^High^ cells at various densities and assessing ALDEFLUOR after 2 days. Our findings indicate that cells cultured in vitro lose the distinctive ALDH activity observed at the time of FACS isolation (not shown).

To assess the differentiation potentials of ALDH^High^ and ALDH^Low^ adventitial cells, FACS-sorted cells were placed in osteogenic or adipogenic differentiation culture conditions. After 14 days, ALDH^High^ adventitial cells showed strong mineralization as assessed by alizarin red staining (Fig. 2B) and active adipocyte formation as shown by Oil Red O staining (Fig. 2C). To further document intrinsic differentiation potentials, we analyzed in cultured cells genes related to osteogenesis and adipogenesis. Genes involved in osteogenesis (Fig. 2D), such as *COL1A1*, *BGLAP, SMAD2,* and *ALPL* among others were enriched in the ALDH^High^ fraction. Similarly, the adipogenesis-related genes *PLIN1*, *APOE,* and *ADIPOR2* were more expressed in the adventitial cell fraction with high-ALDH enzymatic activity (Fig. 2E). However, other expressed genes related to differentiation toward bone and fat, respectively, were enriched in the ALDH^Low^ subset, highlighting the complexity of gene expression predicting functionality. Nonetheless, all these data suggested differences in the predisposition of the ALDH^High^ adventitial cell subset for mesenchymal cell lineages, which was previously corroborated by in vitro differentiation assays. In addition, the effect of ALDH inhibition on ALDH^High^ cells was assessed. Treatment with the ALDH inhibitor DEAB leads to an increase in osteogenic and adipogenic potentials, as well as decreased cell proliferation (Supplementary Fig. S1). We then determined by flow cytometry the expression by ALDH defined adventitial cells of the canonical MSC markers CD90, CD105, CD44, and CD73, showing that adventitial cells with high- and low-ALDH activity express MSC markers at similar levels, as compared to adipose derived MSCs (Fig. 2F). Altogether, these results suggest that ALDH^High^ adventitial cells are enriched in high-proliferation cells primed for mesodermal differentiation.

### Transcriptomes Confirm That High-ALDH1A1 Activity Is a Marker of Mesenchymal Early Progenitors in the Tunica Adventitia

**Figure 3.**
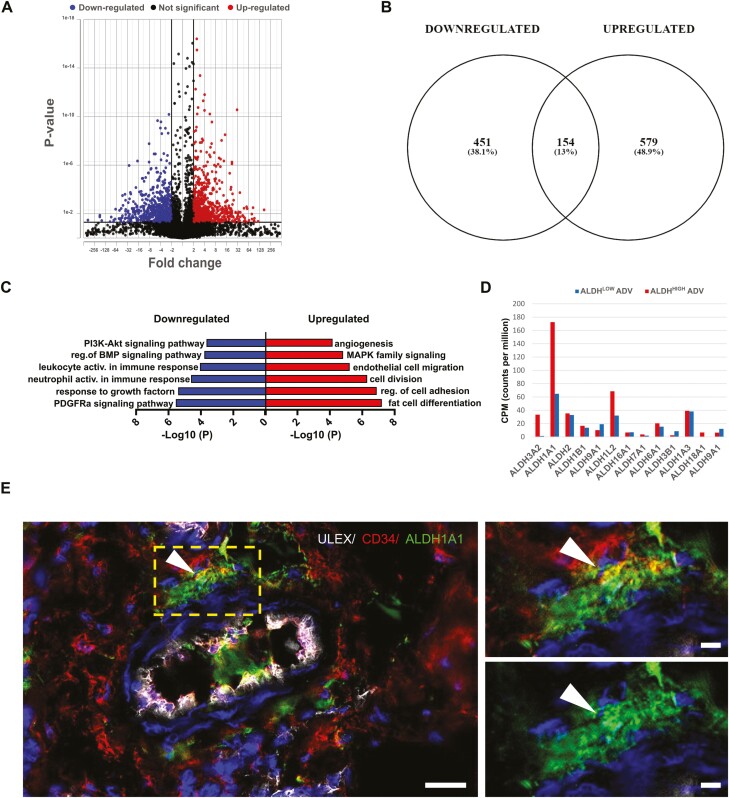
Transcriptome analysis reveals mesenchymal stem cell features in ALDH^High^ adventitial cells. (**A**) Genes differentially expressed in the ALDH^High^ subset of adventitial cells. Significance threshold was set as fold change higher than 2 and *P* value below .05. (**B**) Venn diagram showing genes significantly upregulated and downregulated, as well as shared expressed genes between ALDH subsets. (**C**) Pathway enrichment analysis of ALDH^High^ and ALDH^Low^ adventitial cell subsets. (**D**) Distribution of ALDH isoforms in ALDH subsets of adventitial cells, indicating that ALDH1A1 is enriched in the ALDH^High^ population. (**E**) Immunofluorescence staining for the Ulex europaeus lectin (endothelium), CD34 (adventitia, endothelium) and ALDH1A1 shows coexpression of CD34 and ALDH1A1 in the tunica adventitia of a blood vessel in adipose tissue (arrowheads). Scale bar = 20 μm (insets = 5 μm).

We sequenced RNA from freshly sorted adventitial cell subpopulations with low- or high-ALDH activity (Fig.1B). Distribution of genes expressed differently—or not—between ALDH^High^ and ALDH^Low^ cells is seen in Fig. 3A. Five hundred and seventy-nine genes were significantly upregulated, 541 were downregulated, and 154 exhibited similar expression between the 2 subsets (Fig. 3B). Genes differentially expressed (*P*<.05, Supplementary Table 1) were analyzed to deduce GO/KEGG-term enrichment.^43^ Cell division, angiogenesis, and MAPK signaling, among others, were more represented in the ALDH^High^ subset, suggesting a proliferative and pro-angiogenic cell population (Fig. 3C). This subset was also enriched in transcripts associated with adipocyte differentiation, confirming its inclusion of adipogenic-committed progenitors, as suggested by the results of culture assays (Fig. 2C). On the other hand, transcripts characteristic of the ALDH^Low^ subset included HIF-1 signaling, PDGFRα expression, BMP regulation, and immune activation (Fig. 3C) highlighting functional differences among subsets of adventitial cells. Interestingly, analysis of BMP signaling showed an enrichment of pro-osteogenic molecules such as BMP4 in ALDH^High^ adventitial cells, confirming the pro-osteogenic phenotype of these cells (Supplementary Fig. S2). Together, these observations suggest that higher ALDH activity marks a progenitor cell subset in blood vessel walls with strong proliferation and differentiation potentials, prone to sustaining angiogenesis and tissue regeneration (Fig. 2).

We used the same RNA libraries to determine which ALDH isoforms are represented in the ALDH^High^ compartment of adventitial cells and observed that ALDH1A1 transcripts are more abundant in this subset (Fig. 3D). Immunohistochemistry with an antibody against ALDH1A1 confirmed the presence of this isoform in CD34+ adventitial cells (Fig. 3E). The same CD34+ ALDH1A1+ adventitial cell subset was detected in human fetal heart and adult uterus, the only other organs tested so far (Supplementary Fig. S3A and S3B). We also stained mouse tissue sections with an anti-ALDH1A1 antibody and found expression almost restricted to the *tunica adventitia* (Supplementary Fig. 3C), suggesting the presence of the same functional cell population in mice. Altogether, these observations bring evidence in favor of the presence of ALDH1A1^High^ mesenchymal progenitor cells in human and mouse vascular walls.

**Figure 4.**
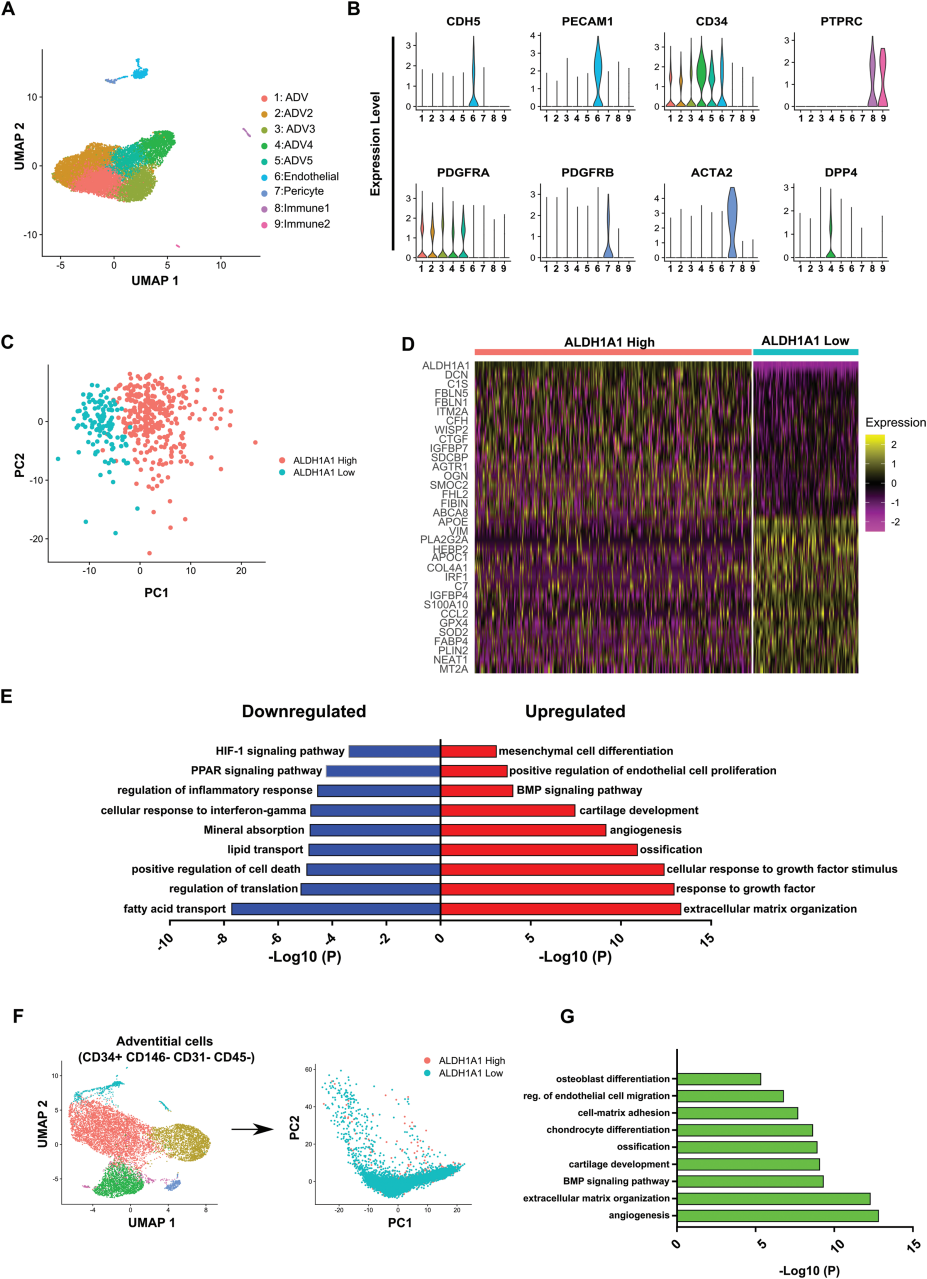
Single-cell RNA sequencing demonstrates that ALDH1A1 expression is correlated with stem cell features in human and mouse tissues. (**A**) UMAP representation of cell compartments in the stromal vascular fraction, including perivascular cells and immune cells. (**B**) Cell type markers used to identify cell clusters. Endothelial cells: CHD5, PECAM1, and CD34; immune cells: PTPRC (CD45); pericytes: ACTA2 and PDGFRβ; adventitial cells: CD34 and PDGFRα. (**C**) Principal component analysis showing the clustering of single cells with high or low expression of ALDH1A1. (**D**) Top 17 differentially expressed genes between ALDH1A1 subsets of adventitial cells. (**E**) Biologic processes represented in ALDH1A1^High^ and ALDH1A1^Low^ adventitial cells, as assessed in Metascape by querying differentially expressed genes. (**F**) Clustering of mouse adiposederived adventitial cells (CD34+ CD146− CD31− CD45−) and subsequent segregation between high and low ALDH1A1 expression. (**G**) Biological processes more represented in the ALDH1A1^High^ subset of mouse adventitial cells.

To further investigate the identity of ALDH1A1+ cells in the *tunica adventitia*, we analyzed a single-cell RNA sequence library (11 144 cells) previously generated from the adipose tissue SVF of a single human donor.^42^ A UMAP projection revealed the presence of 9 cell clusters: one of endothelial cells (expressing *PECAM1* and *CDH5*), 2 of immune cells (expressing *PTPRC*), one of pericytes (expressing *ACTA2* and *PDGFRβ*), and 5 clusters of adventitial cells, expressing CD34 and PDGFRα and comprising 94% of the total cells (10 579 cells). CD34^+^PDGFRα^+^ adventitial cell clusters were subset and further subdivided based on high and low/neg *ALDH1A1* expression on a normalized scale of 0 to 4 (high: above 2.5; low: less than 1) accounting for 329 and 163 cells, respectively. Principal component analysis demonstrated clear separation between *ALDH1A1*^High^ and *ALDH1A1*^*L*ow^ cells (Fig. 4C). Clustering of single cells showing the top 17 genes differentially expressed between ALDH1A1 subsets illustrated clear transcriptome differences (Fig. 4D). Further analysis of differentially expressed genes revealed biologic mechanisms characteristic of ALDH1A1^High^ adventitial cells, as compared to ALDH1A1^Low^ counterparts (Fig. 4E). These comprise extracellular matrix regulation, angiogenesis, cartilage development, ossification, and mesenchymal cell differentiation. Moreover, BMP and ERK signaling are more represented in this subset, confirming previous results and expanding our understanding of this cell fraction. It is worth noting that other isoforms of ALDH also contributed to the ALDH^High^ fraction in the ALDEFLUOR assay, which suggests that isoforms other than ALDH1A1 also play roles in mesenchymal progenitors.

Because we also detected ALDH1A1 expression in the adventitia of mouse blood vessels (Supplementary Fig. 1C), we reanalyzed single-cell RNAseq data from the SVF of mouse adipose tissue.^42^ We identified several clusters with the adventitial cell immunophenotype that mimic the human counterpart (CD34^+^, PDGFRα^+^, CD31^−^, CD45^−^, PDGFRβ^−^). Clusters of other cell types including pericytes and endothelial cells were removed. We further stratified these mouse adventicyte-like cells by expression of high/low ALDH1A1 (Fig. 4F) and performed GO/KEGG-term enrichment analysis (Fig. 4G). Among the processes and mechanisms over-represented in mouse ALDH1A1^High^ adventitial cells, we retrieved those detected in their human counterparts: osteoblast differentiation, BMP signaling, angiogenesis, and extracellular matrix organization. This further suggested the phylogenetic conservation of perivascular mesenchymal progenitor cells expressing and activating ALDH1A1.

## Discussion

Blood vessel walls contain mesenchymal progenitors that have been principally characterized once turned into mesenchymal stem cells in long-term culture. As concluded from experiments in reporter mice, perivascular cells can play a role in the turnover and regeneration of some cells and tissues, although the extent to which such ubiquitous progenitors contribute to organ homeostasis and repair, as well as vessel remodeling, fibrosis, and other pathologic alterations is not known. The outmost *adventitial* layer of arteries and veins is still now seen as a niche for diverse lineage committed progenitor cells, some of which were recently identified on expression of transcription factors and surface markers.^8,13-16,18,20,41,44^ Here, we demonstrate using combined cellular and molecular approaches that higher ALDH native activity in perivascular adventitial cells is associated with genuine mesenchymal stem/progenitor cell features once these cells have been established in culture. Conversely, ALDH^Low^ cells exhibit modest MSC potential in vitro. Altogether, these results demonstrate that high-ALDH activity typifies MSC progenitors in the perivascular adventitial cell niche. We also observed that ALDH1A1 is the main isoform present in the adventitia, but the participation of others in the observed enzyme activity cannot be excluded. Importantly, these data indicate that the previous description of a subpopulation of strongly regenerative ALDH^High^ cells among conventional, culture-derived MSCs^45^ reflects the existence of a related tissue resident cell, and further support a developmental affiliation between adventitial cells and cultured MSCs.^4^

Compelling evidence has documented ALDH activity as a key factor in normal and cancer stem cells. Isoforms of ALDH regulate gene expression, cell differentiation and proliferation, and stem cell activity.^46^ ALDH activity on cell signaling has been documented in tumor endothelial cells^47,48^ and cancer stem cells.^22,24,28,29,31,35,43,49-51^ Mechanisms underlying the association between ALDH activity and cell “stemness” in cancer include Wnt/β-catenin^52,53^ and HIF1α/VEGF signaling.^39^ In the context of mesenchymal progenitors, a study in the Yan Yellow breed of Chinese cattle showed that over-expression of ALDH1A1 in MSCs/pre-adipocytes drives higher adipogenic differentiation.^54^ Conversely, a study in mice by the same group showed that inhibition of ALDH1A1 prevents diet-induced obesity,^55^ which suggests that ALDH1A1 inhibition targets progenitor cells in adipose tissue and corroborates, to a degree, our observations on human ALDH^High^ cells showing high-adipogenic potency.

The fraction of CD34^+^ adventitial cells (approximately 15%) displaying the highest clonogenic and differentiation potentials in vitro are all ALDH^High^, which further supports the concept of a functional heterogeneity within innate mesenchymal progenitors, correlated with the intensity of ALDH activity. Capoccia et al showed that bone marrow-derived mononuclear cells with high-ALDH activity are better at improving reperfusion in mouse ischemic hind limbs, even at low numbers.^56^ Likewise, HSCs (CD34^+^Lin^−^) with high-ALDH activity have higher bone marrow reconstitution capacity, in comparison to their low-enzymatic activity counterparts.^57^ It is, therefore, increasingly clear that ALDH activity may help identify developmental hierarchies of regenerative—but also malignant—cells in different tissues, and we now extend this principle to tissue-resident mesenchymal progenitor cells, the forerunners of MSCs.

MSCs are popular cell therapy tools.^58-61^ However, incompletely understood modes of action and variable clinical outcomes have made it difficult to get the FDA to approve MSC-based treatments. Among the reasons for this inconsistency are variations between human donors and organs used as sources, cell heterogeneity of the starting material, modifications induced by culture, and lack of a fully defined MSC immunophenotype. The use of milder tissue dissociation and uncultured cells may palliate some of these issues. For instance, we showed that non-enzymatic tissue dissociation leaves the perivascular niche intact and in turn increases the release of soluble factors mediating tissue regeneration.^62,63^ Alternatively, MSCs grown in culture from a purified subset of adventitial cells with high-ALDH activity may have higher, more consistent regenerative capacities than those derived from total cell populations. In a more “translational” perspective, this may represent the main interest of the present results, although it will be essential to verify that cultured ALDH^High^ adventitial progenitors possess the other canonical properties of MSCs: immunomodulation and secretion of other cytokines and growth factors.

Importantly, while the ALDEFLUOR reagent is metabolized by some preferred isoforms of ALDH, this demarcation is not strict, which makes the assay less specific.^35,64^ For instance, ALDH7A1 contributes to the fraction of cells with high-ALDH activity in prostate cancer, whereas ALDH3B1 is highly expressed in breast and lung tumors (reviewed by Marcato et al 2011). This raises questions regarding adventitial cells where other ALDH isoforms are active. Moreover, we showed that inhibition of ALDH in vitro leads to more bone and fat formation, which may indicate more complex mechanisms of ALDH regulation during mesodermal differentiation.

In conclusion, we demonstrate that high-ALDH activity in vascular adventitial cells is directly related to the ability of these cells to yield mesenchymal stem cells in culture. We propose ALDH1A1 as a novel, more restrictive marker for innate, tissue-resident mesenchymal stem cells, which could be used to derive MSCs with consistently higher tissue regenerative potential.

## Supplementary Material

szad024_suppl_Supplementary_Figures

## Data Availability

Single cell RNA sequencing data have been published and deposited with the accession number: GSE128889. All other data are available upon request.
