# Supplementary material for: Aldehyde Dehydrogenase, a Marker of Normal and Malignant Stem Cells, Typifies Mesenchymal Progenitors in Perivascular Niches
Source: Stem Cells Transl Med. 2023 Jun 1;12(7):474–84. doi: 10.1093/stcltm/szad024 (PMC10651226; doi:10.1093/stcltm/szad024)
Supplement: szad024_suppl_Supplementary_Figures [file szad024_suppl_supplementary_figures.pdf]

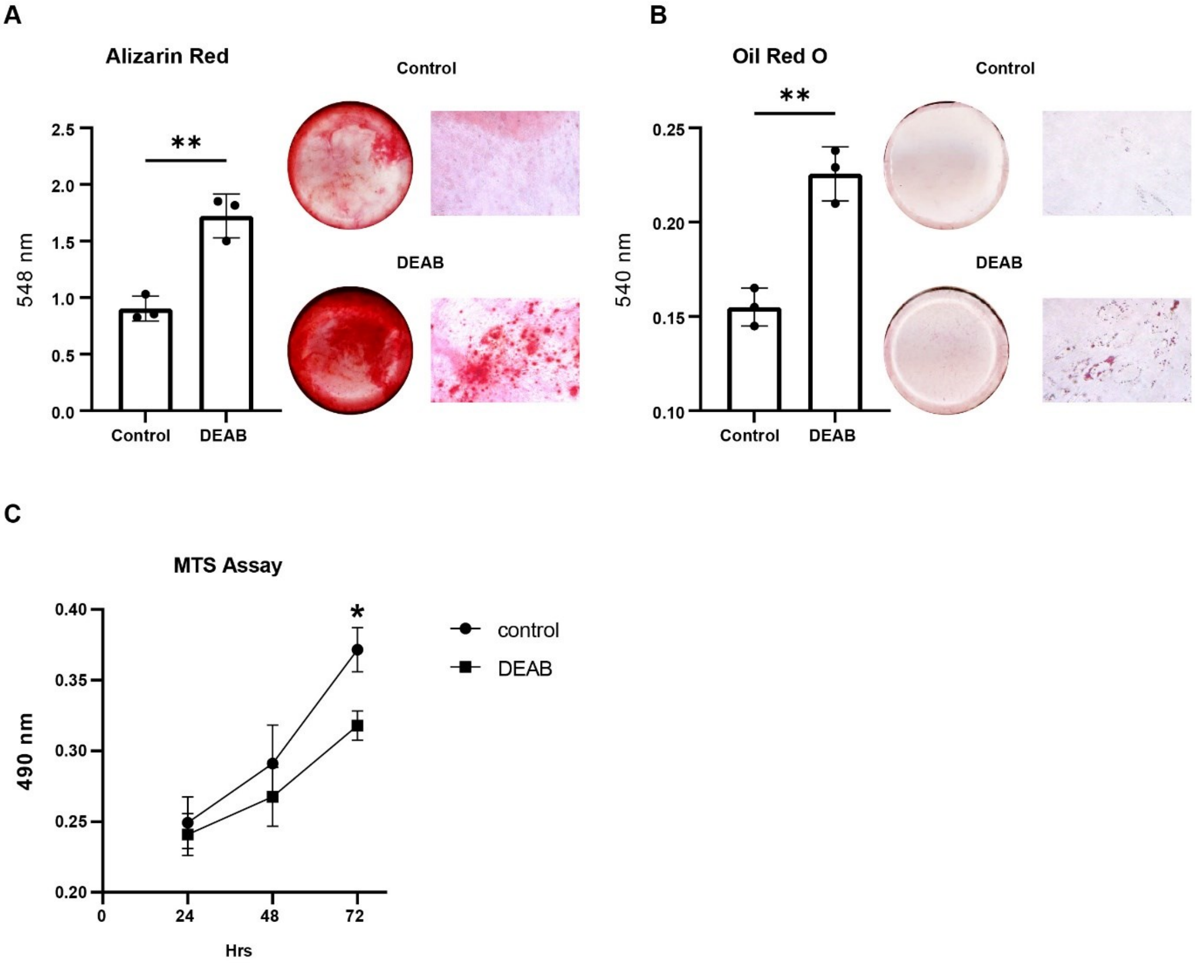

Supplementary Figure 1. Effect of ALDH inhibition on the differentiation and proliferation of ALDH^{High} adventitial cells. (**A-B**) Mesodermal differentiation of ALDH^{High} adventitial cells upon ALDH inhibition with DEAB. (**A**) Osteogenic potential assessed by Alizarin red staining, and measured by photometric quantification of alizarin red . (**B**) Adipogenic potential assessed by Oil red O staining and measured by photometric quantification of Oil Red O . (**C**) Cell proliferation measured by the MTS assay at different time points with or without ALDH inhibition. Unpaired t-test was used for two-group comparisons. Two-way Anova was used to analyze proliferation. Data are shown as mean ± SD. n=3. * $P \leq 0.05$. ** $P \leq 0.01$.

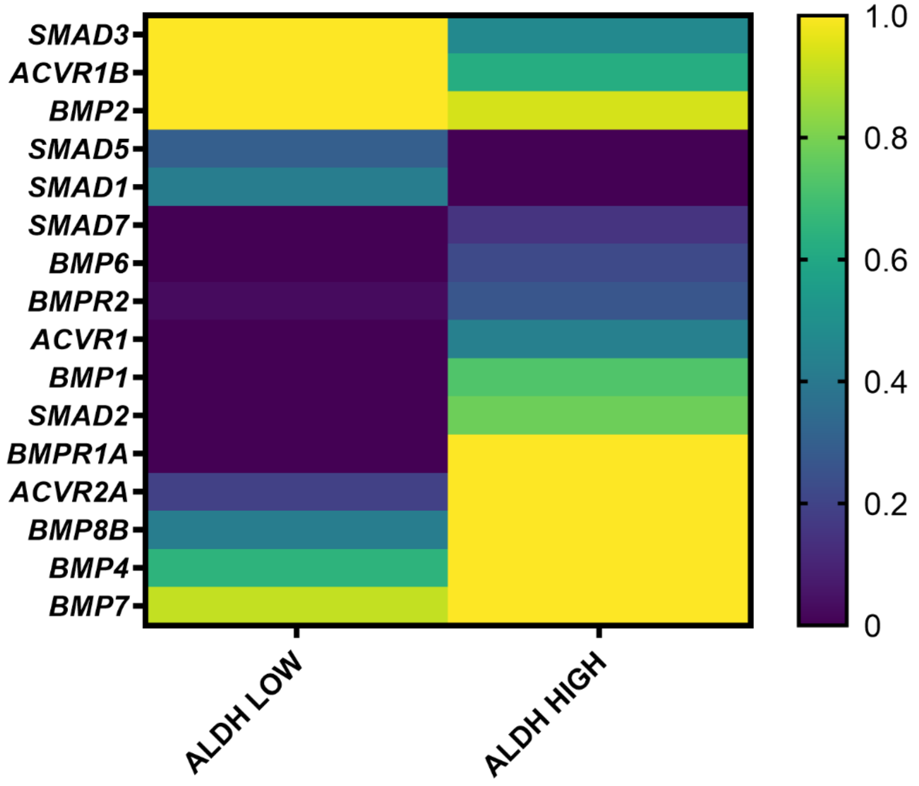

Supplementary Figure 2. Gene expression of BMP related molecules. Heatmap of BMO ligands, receptors, and downstream targets in ALDH subsets of adventitial cells.

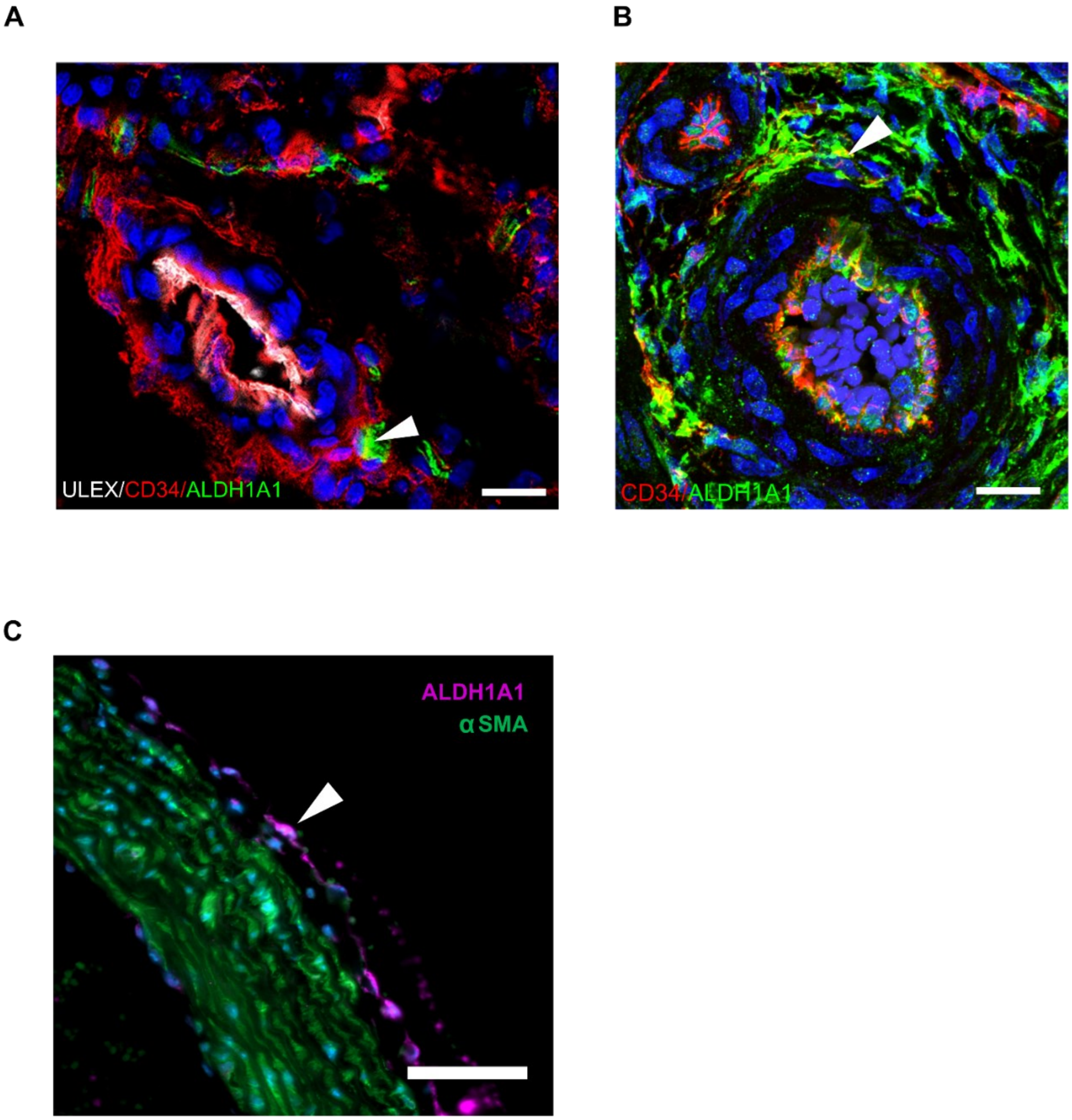

Supplementary Figure 3. ALDH1A1 expression is conserved across organs and species. Immunofluorescence detection of the *Ulex europaeus* lectin (endothelium), CD34 (adventitia, endothelium) and ALDH1A1 shows co-expression of CD34 and ALDH1A1 in the tunica adventitia of blood vessels of **(A)** human fetal heart, **(B)** human adult uterus, and **(C)** mouse cardiac tissue (arrow heads). Scale bars: 20 µm (A), 20 µm (B) and 200 µm (C).
